# App-enhanced transdiagnostic CBT for adolescents with mood or psychotic spectrum disorders

**DOI:** 10.1016/j.jad.2022.05.094

**Published:** 2022-05-18

**Authors:** Marc J. Weintraub, Megan C. Ichinose, Jamie Zinberg, Monica Done, Georga M. Morgan-Fleming, Catherine A. Wilkerson, Robin D. Brown, Carrie E. Bearden, David J. Miklowitz

**Affiliations:** a Semel Institute for Neuroscience and Human Behavior, University of California, Los Angeles, USA; b Department of Psychology, University of California, Los Angeles, CA, USA

**Keywords:** cognitive behavioral therapy, youth, depression, unipolar, bipolar, clinical high risk, psychosis, Unified Protocol, mHealth

## Abstract

**Background:**

Although transdiagnostic forms of cognitive-behavioral therapy (CBT) have been evaluated in individuals with depressive and anxiety disorders, few studies have examined their suitability for more severe disorders, such as recurrent or persistent major depressive disorder, bipolar disorder, or psychotic spectrum disorders. This study examined the acceptability and initial efficacy of an app-enhanced Unified Protocol for Adolescents [UP-A] when including youth with more severe mood disorders or psychotic spectrum disorders.

**Methods:**

We first adapted a mobile application (app), based on user-centered feedback from adolescents and their parents, to assist participants in reviewing session content, practicing skills learned in previous treatment sessions, and monitoring symptomatic progress. A total of 24 adolescents (*M* age = 15.2 years, *SD* = 1.6) with mood or psychotic spectrum disorders and their parents then participated in an open trial of the app-enhanced group treatment given over 9 weekly sessions.

**Results:**

Adolescent participants and their parents rated the group treatment and mobile app as acceptable and useful. We observed significant improvements over the 9-week treatment in adolescents’ depressive symptoms, attenuated psychotic symptoms, and global functioning. The frequency with which adolescents used the mobile app between sessions was positively related to symptomatic and functional gains.

**Conclusions:**

Initial findings suggest the acceptability and feasibility of a mobile app that enabled adolescent participants and their parents to review session content and practice treatment skills. Findings also indicated improvements in psychiatric and functional outcomes among the adolescent participants over the course of the app-enhanced treatment. Randomized clinical trials are needed to evaluate the efficacy of app-enhanced CBT in improving symptoms and functioning in adolescents with mood or psychotic spectrum disorders.

## 1. Introduction

There is a substantial need for effective and inclusive psychosocial treatments for youth with severe mood (i.e., recurrent or persistent major depressive disorder and bipolar spectrum disorders) and psychotic spectrum disorders. Various psychosocial treatments, including cognitive-behavioral therapy (CBT) and family-focused treatment have shown efficacy in reducing mood and psychotic symptoms in youth with these conditions (Addington et al., 2011; French, 2014; Miklowitz et al., 2014; Miklowitz et al., 2017; Pfennig et al., 2014). However, psychosocial treatments have traditionally been developed as disorder-specific treatments. These “siloed” treatment manuals have posed significant limitations for the treatment of youth with more severe mood disorders and psychotic spectrum disorders, as many providers feel ill-equipped to treat patients with these symptoms and many families struggle to find treatment for their child (McGorry and Nelson, 2016).

Despite certain symptomatic differences, there are overlapping cognitive and behavioral processes shared by individuals with psychosis and severe mood disorders and those with less severe mood and anxiety disorders (McGorry et al., 2018). Notably, adolescents with psychotic disorders frequently have comorbid depressive disorders, and adolescents with mood disorders often have comorbid anxiety disorders (Addington et al., 2017; Duffy et al., 2018). There is also uncertainty of prognosis in high-risk youth, as the majority of youth who have subclinical (“at risk”) symptoms of bipolar or psychotic disorders do not go on to develop these conditions (Fusar-Poli et al., 2012). Thus, there has been a significant push for transdiagnostic treatment approaches (Berk et al., 2007; Fusar-Poli et al., 2014; McGorry and Nelson, 2016).

One such transdiagnostic treatment that can be used to include youth with more severe mood disorders and psychotic spectrums disorders is the Unified Protocol for Adolescents (UP-A; Ehrenreich-May et al., 2017a). We have previously provided rationale for use of UP-A in this population, and also describe some of the modifications made to the treatment for its delivery in a shortened group format (Weintraub et al., 2020). The UP-A was originally developed as a transdiagnostic CBT for emotional disorders (anxiety and depressive disorders; Ehrenreich et al., 2009). The psychological principles of the UP-A are based on the shared etiology, co-occurrence, and overlapping emotional, cognitive, and behavioral processes within emotional disorders (Ehrenreich-May and Bilek, 2012; McHugh and Barlow, 2010). To date, individuals with more severe mood disorders and psychotic spectrum disorders have not been included in studies investigating the efficacy of the UP.

One significant limitation in our piloting of the UP-A for youth with more severe mood and psychotic spectrum disorders were the low rates of homework completion, such as thought monitoring and behavioral activation exercises (Weintraub et al., 2020). CBT’s mechanisms of therapeutic change hinge upon practice and implementation of the treatment tasks (Beck, 1979; Kazantzis et al., 2010). Variable rates of treatment adherence are a significant limitation of CBTs, as the rates of homework adherence in adolescents average about 50% across sessions and typically decline over the course of treatment (Gaynor et al., 2006; Simons et al., 2012). To address this therapeutic challenge, our group has sought to improve treatment adherence and clinical outcomes via mobile applications as adjuncts to psychotherapy among youth with mood disorders (Miklowitz et al., 2021). Mobile health (mHealth) technologies have helped increase adherence to treatment tasks (e.g., skill-practice) in adolescents with mood and anxiety disorders (Pramana et al., 2018; Wilansky et al., 2016). mHealth applications (apps) can facilitate many components of CBT, including psychoeducation, and the rehearsal of cognitive skills (Aguilera and Muench, 2012). Applications may also incorporate features designed to increase adherence to treatment tasks (e.g., text reminders) and make engagement with homework exercises more acceptable and engaging.

In the present study, we sought to adapt an existing mobile app (Miklowitz et al., 2021) for use with our modified group-based UP-A to target three components of patients’ adherence to treatment: skill-practice, symptom monitoring, and review of session content. We held focus groups with participants who had taken part in previous CBT groups to identify core clinical and design features to include in a revised mobile app. We then conducted an open trial of the app-enhanced treatment over 9 weekly group sessions for adolescents ages 13–17 years with mood or psychotic spectrum disorders and their parents. We aimed to determine the acceptability of the app-enhanced group treatment through user ratings of the app and frequency of usage over the course of the two-month treatment. Finally, we examined the association between the app-enhanced treatment and improvements in psychiatric and functional outcomes, and the association of app usage with these outcomes.

## 2. Methods

### 2.1. Study overview

In the first phase of this study, our research team adapted a mobile app that was developed for use in family-focused therapy (FFT) for adolescents with mood disorders (Miklowitz et al., 2021). We began by modifying the content and the skills to be consistent with the skills presented in the UP-A (Ehrenreich-May et al., 2017a). Semi-structured focus groups were held prior to and following the first app-enhanced group with both youth and parents. Youth and parent participants from the following three group cohorts provided feedback on the app in their post-treatment assessments. The app was iteratively updated throughout the open trial to fix bugs and improve the user experience.

In the second phase of this study, we conducted a 9-week trial of the app-enhanced UP-A for adolescents with mood or psychotic spectrum disorders and their parents across four group cohorts (each containing 6 to 8 participants). All participants received nine sessions of group treatment, with assessments of psychosocial functioning at pre-treatment and post-treatment. Primary outcome measures included acceptability of the treatment, user experience, and adherence to the mobile app. Secondary outcomes included measures of youths’ symptomatic and functional changes, as well as the relationship between app usage and these psychosocial outcomes over the two-month treatment.

### 2.2. Participants

Youth were recruited primarily from two specialty outpatient programs at UCLA Semel Institute – the Child and Adolescent Mood Disorders Program and the Center for the Assessment and Prevention of Prodromal States. Participants had to meet for a lifetime DSM-5 unipolar or bipolar mood disorder (American Psychiatric Association, 2013), including major depressive disorder, persistent depressive disorder, bipolar mood disorder or unspecified depressive or bipolar disorder based on semi-structured interview using the MINI International Neuropsychiatric Interview for children and adolescents (Sheehan et al., 1998; Sheehan et al., 2010) and/or current criteria for the psychosis-risk syndrome based on the Structured Interview for Prodromal States (Miller et al., 2003). Participants had to be aged 13 years, 0 months to 17 years, 11 months, fluent in English, able to attend and appropriately participate in all treatment sessions, and have at least one parent willing to join the treatment. All participants were also required to have access to a smartphone.

We excluded participants who were too acutely ill to participate in treatment in an outpatient setting and/or who would be disruptive to the other group participants. Specfically, participants with significant behavioral issues (e.g., aggression, oppositionality), active suicidality, substance abuse, or acute psychotic symptoms that would interfere with the treatment or require more intensive treatment were excluded from this study and referred elsewhere.

### 2.3. Intervention

The treatment delivered in this study is a modified version of the Unified Protocol (UP) for Adolescents – a CBT protocol for adolescents with mood and/or anxiety disorders (Weintraub et al., 2020). The treatment includes nine 90-min weekly group sessions delivered in three modules – psychoeducation, cognitive skills, and behavioral skills. The psychoeducation module involved identifying areas of difficulties and goals, brainstorming reasons to make a change in behavior, learning about the functions of emotions, and communicating one’s emotions. Cognitive skills included thought monitoring, identifying thinking traps (i.e., cognitive distortions), and detective thinking (i.e., cognitive reappraisal). Behavioral skills included various relaxation techniques, opposite action, and pleasant events scheduling. See Table 1 for a breakdown of the sessions and their contents.

Adolescents and at least one parent participated in the treatment. To encourage open disclosure and to promote greater cohesion among group members, the first ~75 min of each 90-min treatment session was delivered to adolescents and parents separately. The adolescents were delivered the treatment in a “teen room,” while at the same time the parents were delivered the treatment in the “parent room.” Parents and adolescents were given the same treatment content and taught the same skills. In the final 10–15 min of each session, the parents and adolescents joined together to discuss the skill(s) of the week, make a plan for how/when they would practice the skill(s), and share this plan with one of the group facilitators prior to ending the session.

### 2.4. Objectives of the mobile application (app)

Youth and their self-identified primary parent were provided unique login access to the app. The app was designed to help the youth and parent accomplish three tasks – review the content of the treatment sessions, practice skills taught in the treatment, and log/review their current symptoms and functional status (also known as the symptom check-in). The app was meant to be used as a direct tool in the therapy session. Clinicians would showcase the treatment materials and have the participants practice the treatment skills in sessions. Additionally, the app served as a method to bridge sessions by allowing participants to review session content and practice treatment skills from their mobile device. The app was accessible via a URL as a mobile-friendly link. Thus, the app could be used on any internet-accessible device, including mobile devices, tablets and personal computers. Participants were asked to practice their treatment skills at least twice per week and perform a symptom check-in at least once per week.

The app included a “provider” login (called the “provider portal”) that was used by treating clinicians between each session to see which skills the participants practiced and the results of their symptom check-ins. Clinicians were also able to set the “skill of the week” that presents on each participan’s homepage of the app.

Two focus groups were conducted, one at the outset of the study and one following the first iteration of the app-enhanced group treatment. Participants in the first focus group had previously participated in one of our UP-A groups during the development phase of the study (Weintraub et al., 2020). In this first focus group, adolescent and parent participants were shown the mobile app developed for youth with mood disturbances receiving family-focused therapy (Miklowitz et al., 2021). The focus group participants were asked predefined questions regarding the user experience (e.g., the flow of the app) and app design (e.g., aesthetic recommendations). They were also asked to provide open-ended feedback regarding the group treatment, including its format, the ordering of sessions, the skills taught in sessions, and barriers to completing treatment skills. All of the data collected from the focus groups were qualitative. With this first focus group’s set of recommendations as guideposts, our research team modified the mobile app for use in a group treatment with the UP-A by creating a module for each of the treatment skills (deep breathing, identifying emotions, thought monitoring, thinking traps, detective thinking, mindfulness, opposite actions, and pleasant activities).

After the first cohort (in which eight participants finished the app-enhanced treatment), we held a second focus group where we again asked predefined questions about user experience and design. Open-ended feedback was also regarding recommendations related to the UP-A treatment skills and app content. We then made a second round of modifications to the app. The app modifications made following each focus group represented the largest changes made to the app; however, additional bug fixes and minor edits were made to the app throughout the open trial when needed.

### 2.5. Study outcomes

We first outlined the main findings from the focus groups and the subsequent app modifications. We then examined the primary outcome of the open trial – participants’ engagement with the treatment via the mobile app and acceptability of the mobile app itself. Engagement was measured by the total number of app interactions, separated as either skill practices (e.g., thought monitoring, opposite action) or symptom check-ins. Participants were asked to practice treatment skills at least twice per week and to do a symptom check-in once a week over the 9-week treatment. App acceptability was rated by the participants (both youth and parents), which included the app’s ease of use and its usefulness in (1) tracking psychiatric symptoms, (2) reviewing treatment content, and (3) practicing treatment skills. App acceptability across each of these items was rated on a scale of 1–10, with 1 indicating “not at all” acceptable and 10 indicating “very” acceptable.

Secondary outcomes of the open trial included mood symptoms and psychosocial functioning, which were assessed at the baseline assessment (i.e., within two weeks prior to the start of treatment) and post-treatment (i.e., within two weeks following the final group session). Mood symptoms were rated by trained research assessors on the on the Children’s Depression Rating Scale, Revised (CDRS) (Poznanski & Mokros, 1996), which measures depressive symptom severity over the past two weeks. Youth participants also self-rated their attenuated psychotic symptoms on the Prodromal Questionnaire Brief (PQ-B), a 21-item measure of, with items rated from 0 (not present) to 5 (present and strongly agree that the experience was frightening, concerning, or caused problems for the individual) (Loewy et al., 2011).

Youth’s psychosocial functioning was measured by trained research assessors using the Clinical Global Assessment Scale (CGAS; Shaffer et al., 1983), a 100-point measure of global functioning. Youth self-rated functioning using the KINDL (Ravens-Sieberer and Bullinger, 2000), which includes 30 items rated on 5-point Likert scales measuring psychosocial functioning across seven domains: physical well-being, emotional well-being, self-esteem, family, friends, school, and illness. Emotion regulation was rated by youth self-report using the Difficulties with Emotion Regulation Scale (DERS; Gratz and Roemer, 2004), which includes 36 items rated on 5-point Likert Scales.

### 2.6. Data analyses

The average app usage (i.e., skills practiced, check-ins, and their combination) was calculated for both adolescents and parents over the 9-week treatment. To determine whether app engagement changed over the course of the four sequential group treatments as the app was being refined, Kendall’s coefficient of rank correlation was used to examine the ordinal relationship between app use and group number (1 to 4). Analysis of variance (ANOVA) was used to compare the first group’s app use (i.e., prior to the final major app updates) and the remaining three groups’ app use. App acceptability values were averaged across participants based on their post-treatment reports.

Repeated measure mixed effect regression analyses were conducted to determine whether there were improvements in depressive, manic, and attenuated psychotic symptoms and global functioning from the baseline assessment to post-treatment. We then used Pearson correlations to determine whether app usage correlated with changes in mood symptoms and functioning. Baseline values were subtracted from the post-treatment values to derive the change value for each participant. The stronger the negative correlation between app usage and mood symptoms, the greater the improvement; positive correlations between app usage and global functioning indicate greater improvement.

## 3. Results

### 3.1. Focus groups

The first focus group, which included six adolescent-parent dyads, contained participants who had completed the UP-A group treatment but did not use a mobile app. Five of the adolescents were female and one was male (mean age = 15.8); all six parents were mothers. Participants cited three main barriers to completing CBT practices during the 9-week group: (1) forgetting to practice, (2) forgetting what the skill(s) were and how to do them, and (3) experiencing the skills as boring and unrewarding in the moment. Participants unanimously favored the use of text reminders to practice the treatment skills, and that the app included a review and an example of how to complete each skill. However, participants felt that the app couple be streamlined to ease navigation (e.g., shorten the text, have less material on each page, include no more than three pages to navigate through per skill module) and to have the app indicate whether the required skill practices were completed (e.g., an icon or badge signaling successful submission). Participants also suggested incentivizing skill practice with a “team challenge,” which would involve having adolescents within a group cohort randomly broken up into two sub-groups that would compete to have the most skill practices completed over the course of the treatment. The app would be used to indicate participants’ team membership as well as catalogue each group’s total skill practice throughout the treatment.

**Fig. 1.**
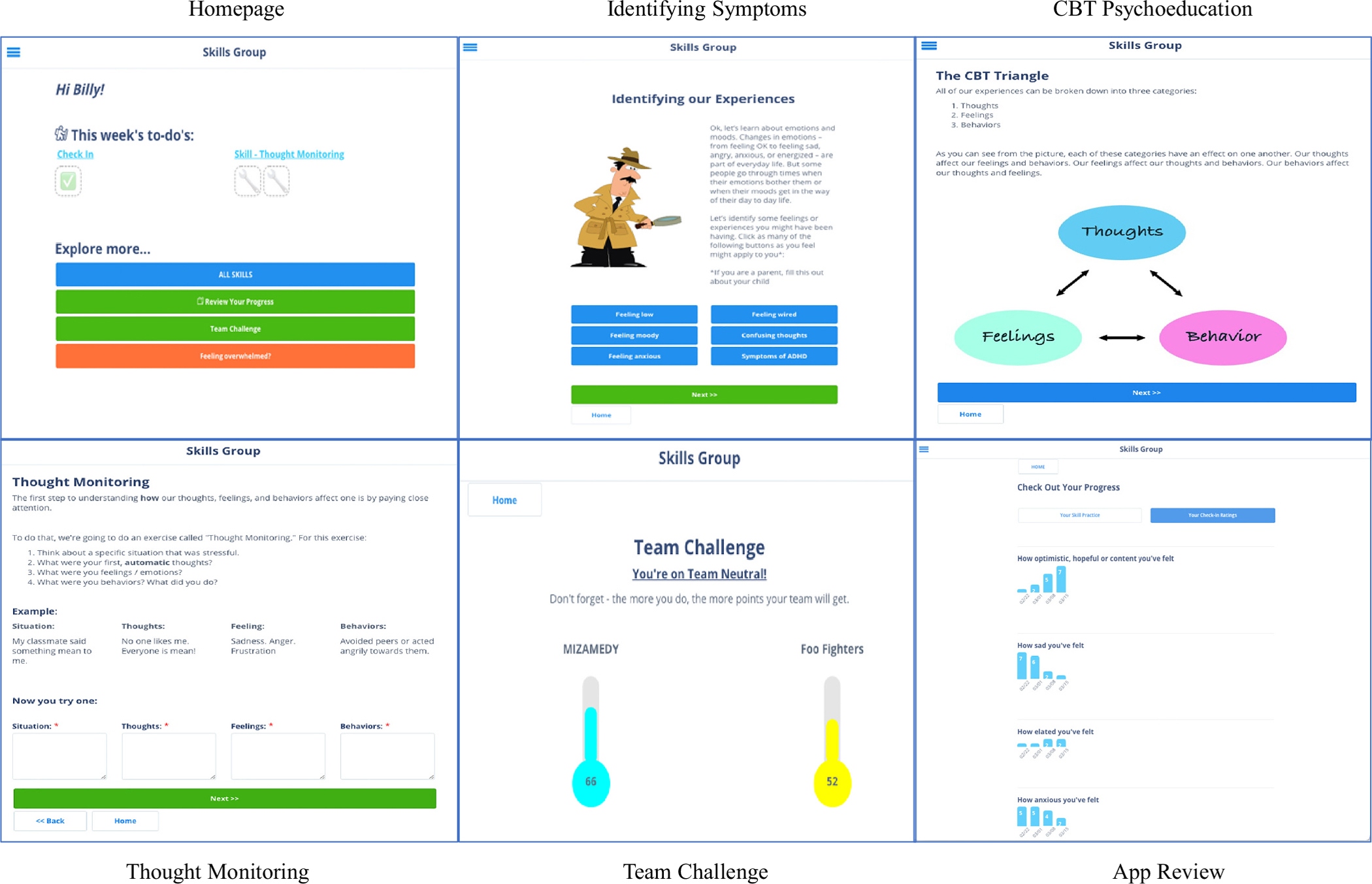
Sample pages of the mobile application.

Feedback from this first focus group guided adaptation of the FFT app for use in our group-based CBT. The homepage was pared down to include (1) a weekly check-in to log psychiatric symptoms, (2) a link to the “Skill of the Week,” and (3) an “Explore More” section. The latter allowed participants to access all of the treatment skills, review their previous skill practice and symptom check-ins, see the Team Challenge points, and access coping strategies if they were feeling acutely stressed. As advised by the focus group, each skill module was set up in three-page blocks – the first page reviews the skill, the second page has an example and space to practice, and the third page allows the participants to see and review their practice. Fig. 1 shows selected screenshots from the app.

The second focus group consisted of three adolescent-parent dyads/triads (mean age = 15.0; one adolescent male, two adolescent females, plus three female and two male parents) who had participated in group-based CBT using the first version of the app. Participants expressed satisfaction with the design and user-interface of the CBT app and appreciated the ability to review each skill and their own skill practice. Overall, participants expressed satisfaction with the app’s brevity of content and simplicity of navigation. Participants reported mixed opinions about the Team Challenge, with some indicating it was motivating and others feeling it was demotivating, particularly if they were using the skills but their other team members were not.

The app was again updated based on feedback from this group. Updates included the ability to personalize app login information, frequency of text reminders, and auto-filling of participants’ ID number on the login screen. A text box was also added to the weekly check-in where participants could journal about how their week went, which could then be reviewed on the “Review your Progress” page of the app. Finally, the Team Challenge feature of the app was changed to a Group Challenge, intending to motivate the whole group to reach certain milestones without having group members compete against each other.

### 3.2. Open trial

A total of 33 youth were assessed for inclusion in four groups, with 31 of those youth initiating treatment. One was excluded due to having active psychosis and the other was no longer interested in the program following the intake assessment. Twenty-four of the 31 youth (77.4%) completed the treatment and post-treatment assessments. Treatment completers attended an average of 7.7 (*SD* = 1.6) of the nine group sessions. See Table 2 for the demographic and clinical characteristics of the youth who initiated the treatment.

### 3.3. App engagement and acceptability

Over the 9-week treatment, the adolescents across all four group cohorts used the app an average of 16.1 times (*SD* = 8.8), including an average of 10.3 (*SD* = 7.5) skill practices and 5.8 (*SD* = 3.3) symptom check-ins. Parents used the app an average of 17.9 times (*SD* = 16.2) over 9 weeks, which included 13.2 (*SD* = 11.8) skill practices and 4.7 (*SD* = 5.0) check-ins. Adolescent total app usage was marginally related to their parents’ total app usage (*r*(23) = 0.38, *p* = 0.07). There were no associations between youths’ app use and demographic variables (i.e., gender, age, ethnicity/race, grade, family income). Measuring the four group cohorts ordinally, there was an increase in skill usage by the adolescents as the groups progressed from the first to the fourth (*r*(23) = 0.48, *p* = 0.003). The same pattern was observed among parents (*r*(23) = 0.72, *p* < 0.001). This increase for the adolescents was most pronounced between the first group (i.e., when the focus groups were held and most of the app modifications were made) and the remaining three groups (*F*(1,22) = 4.51, *p* < 0.05).

**Fig. 2.**
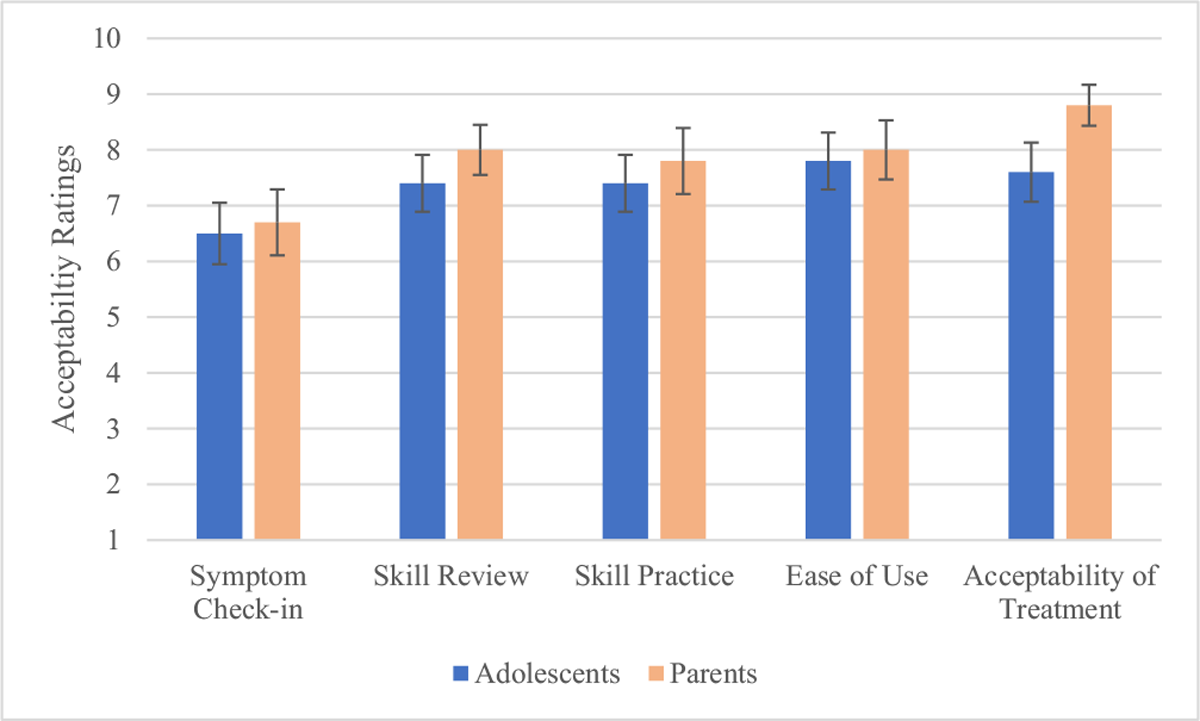
App and Treatment Acceptability Ratings by Adolescent and Parent Participants at Treatment Termination Errors bars represent standard error of the mean.

Overall, the app acceptability items (symptom check-ins, reviewing session content, and practicing skills) were rated positively by both adolescents and parents (Fig. 2). The check-in feature was rated significantly less favorably compared to the session review and skill practice features by both adolescents and parents (*p*s < 0.05). There were no statistical differences in adolescents’ and parents’ ratings of the app. However, parents rated their acceptability of the treatment itself significantly higher than the adolescents (*t*(21) = −2.39, *p* = 0.03). The acceptability rating of the app and treatment are presented in Fig. 2.

### 3.4. Mood and functional outcomes

Adolescents’ depressive symptoms on the CDRS and their CGAS global functioning (prior two weeks) significantly improved over the 9-week treatment (*F*(1,29.8) = 16.60, *p* < 0.001; *F*(1,28.69) = 14.76, *p* = 0.001, respectively). Adolescents’ self-reported reported attenuated psychotic symptoms on the PQ-B also significantly improved from pre-to post-treatment (*F*(1,24.1) = 10.03, *p* = 0.004, respectively). Additionally, adolescen’s ratings of their illness-related functioning on the KINDL and their emotion regulation via the DERS improved from pre- to post-treatment (*F*(1,25.18) = 6.02, *p* = 0.02; *F*(1,22.3) = 4.69, *p* = 0.04, respectively).

### 3.5. Relationships between app use and psychosocial outcomes

There was no relationship between adolescen’s baseline psychiatric symptoms, functioning or demographic variables and app usage over the course of treatment (skills, check-ins, or their combination). However, poorer functioning (as measured on the CGAS) in youth at baseline was associated with greater overall parental use the app (*r*(23) = −0.44, *p* = 0.03).

Adolescents’ frequency of skill practice on the app was related to greater improvements in depressive symptoms on the CDRS and improved functioning on the CGAS from pre- to post-treatment (*r*(23) = −0.55, *p* = 0.005; *r*(23) = 0.44, *p* = 0.03, respectively). Adolescents’ frequency of skill practice on the app was also associated with greater improvements in self-esteem and social/peer functioning as rated on the KINDL (*r*(23) = 0.44, *p* = 0.04; *r*(23) = 0.50, *p* = 0.02, respectively). Interestingly, parents’ skill practice was also associated with their adolescen’s report of improved social relationships on the KINDL (*r*(23) = 0.49, *p* = 0.02) as well as improvements in emotion regulation on the DERS (*r*(23) = 0.44, *p* = 0.04).

Adolescents’ frequency of symptom check-ins was unrelated to symptomatic or functional changes. However, parents’ check-ins of their adolescents’ symptoms on the app were associated with greater improvements during treatment in their youth’s depressive symptoms, as rated on the CDRS (*r*(23) = −0.45, *p* = 0.03).

## 4. Discussion

This study presents the open-trial of an app-enhanced group CBT for youth with mood and psychotic spectrum disorders. Starting with a mobile application developed for family-focused therapy for youth with mood disorders (Miklowitz et al., 2021), we adapted the mobile application to be used in four treated cohorts, with focus groups conducted before and after the first group. Overall, participants found the app to be satisfactory, as indicated by high ratings of acceptability as well as increased use of the app over the four successive cohorts. This study also tested the initial effects of the app-enhanced CBT. Initial findings indicated that the participants showed improvements in depressive symptoms, attenuated psychotic symptoms, and global functioning over the 9-week treatment. Further, increased app usage was associated with increased symptomatic and functional improvements across these domains.

The development of the app, as expected, was an iterative process that continued beyond the focus groups and will continue beyond this open trial. The main suggested modifications that surfaced among participants throughout the development and trialing of the app involved personalization, ease of access, and succinctness. Participants wanted the app to accommodate their individual needs and requests, including personalizing login information and having space to write down ideas in their own words (as opposed to being “boxed” into multiple-choice responses). Participants also wanted to select their own reminders for skill practice and for the reminder texts to include specific messaging that relates to the participant (e.g., “Don’t; forget to go for a *walk* this afternoon as part of your *Opposite Action*”).

Ease of access (i.e., being able to get to and from sections within the app with ease) and succinctness (i.e., brevity of text and modules) were continuous goals throughout the open trial. This included updating the app with various features such as auto-fill for participants’ login information and reducing the amount of text within the app. Participants reported preferring brevity over detail for skill review and practice exercises. Together, paring down the app to make the text and navigation between sections of the app as brief and succinct were key goals of the app development.

Participants were less satisfied with completing the weekly symptom check-ins. The symptom check-ins, which involved making Likert-type ratings to indicate one’s mental health, were perceived to be reductionistic. Additionally, some serious symptoms (e.g., unusual thought content) are not easily described in symptom checklists, as they reflect variable experiences that are difficult for participants to bucket into categories. Moreover, some participants may have disliked being reminded of their symptoms or the severity of their functional impairments on a weekly basis.

Overall, participants practiced treatment skills on the app a little more than once per week over the 9-week treatment (10.7 times, on average). Findings from previous studies suggest that adolescents typically engage in about 50% homework compliance, which would amount to practicing a skill every other week (Clarke et al., 1992; Gaynor et al., 2006). Despite encouragement to practice skills multiple times per week, weekly engagement with the treatment skills in this study still represents about a two-fold improvement from previous trials of standard CBT without the use of an app.

The analyses concerning symptom and functional change must be understood within the context of an uncontrolled trial. Participants showed significantly improved depressive symptoms as well as improvements in clinical and global functioning and emotion regulation. These within-group results parallel previous work with the Unified Protocol (UP) both for adults and adolescents with anxiety and/or depressive disorders (Ehrenreich-May et al., 2017b; Sauer-Zavala et al., 2020). While our initial feasibility study showed pre- to post-treatment numerical improvements in psychiatric symptoms and functioning in a sample of 10 individuals with mood and psychotic spectrum disorders (Weintraub et al., 2020), this study extends these earlier findings by demonstrating statistical improvements in these domains among adolescents with mood and psychotic spectrum disorders who received UP-A. These results provide additional evidence of feasibility for including youth with more severe mood disorders and psychotic spectrum disorders in the UP-A as well as point to the initial positive effects this treatment has for this population. The finding that youths reported better emotion regulation after treatment was encouraging, because this is a theorized mechanism of action for the UP (Ehrenreich et al., 2009; Fairholme et al., 2010). Future studies should examine the efficacy of UP-A compared to a randomized comparison group who receive group CBT without the app, or perhaps the app as a stand-alone treatment.

Notably, app use in both youth and parents was related to improvements in depressive symptoms and functional outcomes. In particular, adolescents’ skill practice and parents’ symptom check-ins were strongly related to improvements in clinical and functional changes over the course of the treatment. Without a randomized control group that did not receive the app, it remains unclear whether the app itself helps to facilitate these practices above what can be accomplished with paper and pencil practices. It has long been believed that outcomes of CBT hinge upon practice of skills, although meta-analyses examining the association between homework compliance and clinical outcomes in youth have been inconclusive (Arendt et al., 2016; Olatunji et al., 2015). Variability in the quality of skill practice may contribute to these inconsistent findings (Kazantzis et al., 2016). It is possible that mobile apps help to improve the quality of the skills practice by providing review of the session content as well as detailed guidance for practicing the skill.

The association of youths’ skill practice and parents’ check-ins on youths’ social functioning was unexpected. Behavioral skills from the treatment (e.g., scheduling pleasant events, opposite action) may have encouraged greater social involvement in the youths’ day-to-day lives. The cognitive skills may have also helped increased youth’s willingness to engage with peers through more helpful, motivating thoughts. While there was minimal focus on emotional communication, the adolescents were encouraged throughout the treatment to share their emotional experiences with family and peers more frequently. Perhaps the treatment led to an expanded emotional vocabulary for adolescents that stimulated more peer engagement. It is also possible that the group format of the treatment that involved peer-to-peer interactions helped improve youths’ willingness to engage with peers outside of the group.

### 4.1. Study limitations

Study limitations include the uncontrolled, nonrandomized design and the sample size. The app was limited in a few key ways. The platform that was used to develop the app, while user-friendly, does not have the sleekness and feature options that characterize many for-profit apps. Participants made clear that the app was not as appealing as mainstream apps (e.g., Instagram, TikTok). Unfortunately, this will likely continue to be a limitation of apps developed solely in the context of research, as researchers cannot move as quickly to make app updates, nor can they devote the same amount of time or money to product development as larger technology firms. Nonetheless, gathering feedback from participants and continuing to make iterative updates to the app will enhance our ability to test the added benefits of mHealth as an adjunct to psychotherapy.

## 5. Conclusion

This study presents the development and open trial of an app-enhanced group-based CBT for adolescents with mood and psychotic spectrum disorders. Initial findings suggest the acceptability and feasibility of a mobile app for use with both adolescents and their parents to review session content and practice treatment skills. Findings also indicated improvements in psychiatric and functional outcomes in these youth over the 9-week treatment. Greater skill practice via the app was associated with greater improvements in the youths’ mood and functional outcomes. By controlling for app exposure in a randomized controlled trial, future research should investigate the app’s effects on skill practice and whether increases in skill practices are associated with enhanced psychosocial outcomes among youth with mood and psychotic spectrum disorders.

## Figures and Tables

**Table 1:** Session-by-session summary of the group-based CBT.

Session	Title	Session content

1	Building and Keeping Motivation	■ Build rapport with adolescents. ■ Discuss key problems and set goals. ■ Determine what motivates the adolescents to change.
2	Emotion Identification Practice. Getting to Know Your Emotions.	■ Provide psychoeducation about different emotions. ■ Discuss the purpose of emotions. ■ Develop the adolescents’ awareness of their feelings. ■ Teach “I” statements to communicate emotions.
3	Linking Emotions to Thoughts & Behaviors	■ Introduce the three parts of an emotion. ■ Introduce the cycle of avoidance and other emotional behaviors. ■ Introduce some common “thinking traps” (i.e., cognitive distortions). ■ Teach the adolescents how to track emotions, thoughts, and behaviors.
4 & 5	Being Flexible in Your Thinking	■ Review common “thinking traps” (i.e., cognitive distortions). ■ Develop the adolescents’ ability to think flexibly about emotional situations. ■ Link thoughts to actions by teaching Detective Thinking and Problem-Solving skills.
6	Awareness of Emotional Experiences	■ Introduce and practice present-moment awareness. ■ Introduce and practice nonjudgmental awareness. ■ Compare and contrast detective thinking and mindful awareness.
7 & 8	Introduction to Emotion-Focused Behavioral Experiments & Situational Emotion Exposure	■ Introduce the concepts of opposite action and emotion-focused behavioral experiments. ■ Discuss the rationale for situational emotion exposures, introduced to the adolescents as another type of behavioral experiment. ■ Conduct sensational exposure exercises to help the adolescents learn to tolerate uncomfortable physical feelings. ■ Brainstorm situational emotion exposures in session and assign exposures for home learning. ■ Engage the adolescents in emotion-focused behavioral experiments for sadness, anxiety, and other emotions.
9	Reviewing Accomplishments and Looking Ahead	■ Review skills and progress toward goals. ■ Create a relapse prevention plan.

**Table 2:** Demographic and baseline illness characteristics of participants in open trial (*N* = 31).

	***Mean (SD)***

**Demographics**	
Youth age (years)	15.1 (1.5)
Primary parent age (years)	49.9 (5.6)
Youth academic grade	10th (1.5)
Household income (selected in ranges)	$100,000 – $149,000
	***n*** (%)
Youth sex, Female	19 (61.3)
Primary parent sex, Female	26 (83.9)
*Youth Racial/ethnic Background*	
White/Caucasian	20 (64.5)
Black or African American	2 (6.5)
Hispanic/Latino	4 (12.9)
Asian or Asian-American	3 (9.7)
Mixed Race	2 (6.5)
	***Mean (SD)***
**Youth Baseline Illness Characteristics**	
Child Depression Rating Scale (CDRS)	44.3 (11.9)
Prodromal Questionnaire-Brief (PQ-B)	22.6 (20.7)
Clinical Global Assessment Scale (CGAS)	46.7 (12.1)
Difficulties with Emotion Regulation Scale (DERS)	106.5 (22.6)
	***n*** (%)
*Primary psychiatric diagnoses*	
Attenuated psychotic syndrome + depressive spectrum disorder	7 (22.6)
Attenuated psychotic syndrome	1 (3.2)
Bipolar I disorder	2 (6.5)
Bipolar II disorder	2 (6.5)
Unspecified bipolar disorder	1 (3.2)
Major depressive disorder	8 (25.8)
Persistent depressive disorder	7 (22.6)
Unspecified depressive disorder	3 (9.7)
*Comorbid diagnoses*	
Attention-deficit/hyperactivity disorder (ADHD)	12 (38.7)
Generalized anxiety disorder	15 (48.4)
Social anxiety disorder	7 (22.6)
Obsessive compulsive disorder	3 (9.7)
Unspecified anxiety disorder	3 (9.7)
*Baseline medications*	
None	12 (38.7)
Antipsychotic	5 (16.1)
Anticonvulsant	8 (29.0)
Antidepressant	20 (64.5)
Anxiolytic	9 (29.0)
Psychostimulant or other ADHD agent	10 (32.3)
